# Conceptualizing Domestic Violence Within Clinical Documentation

**DOI:** 10.1177/23333936241271165

**Published:** 2024-10-14

**Authors:** María Atiénzar-Prieto, Shannon Dhollande, Silke Meyer, Diksha Sapkota, Karen-Ann Clarke

**Affiliations:** 1 University of the Sunshine Coast, Sippy Downs, QLD, Australia; 2 CQUniversity, Brisbane City, QLD, Australia; 3 Griffith University, Meadowbrook, QLD, Australia; 4 Griffith University, Brisbane, QLD, Australia

**Keywords:** intimate partner violence, emergency, mental health, domestic violence, hospital, healthcare

## Abstract

Domestic and family violence (DFV) is a global issue with significant impacts on victim-survivors. The emergency department (ED) serves as the initial point of contact for victim-survivors. Given the significant role that clinical notes play in the management of patients and the communication between healthcare professionals, understanding how healthcare practitioners describe and document abuse reported in emergency settings is crucial. Yet, there remains a gap in understanding how health professional document DFV in the medical records of women presenting to the ED. Therefore, this qualitative descriptive study explored how DFV is documented in patient records of women presenting to the ED. Clinical notes from healthcare workers, including medical practitioners, nurses, social workers, mental health clinicians and ambulance officers, were qualitatively analyzed. Overall, the study included 43 presentations from 32 women (aged 18–56 years old) who visited a regional ED, during which instances of DFV were noted. An inductive content analysis resulted in the identification of four categories, including (a) DFV articulated in direct speech, (b) Unambiguous DFV, (c) Unconfirmed DFV, and (d) Problematic relationship. Although most references to DFV in the clinical notes included direct quotations from the patient’s descriptions of abuse or were documented unambiguously by healthcare professionals, a notable number of clinical notes exhibited a degree of caution or reluctance to acknowledge DFV dynamics when describing these events. These findings support the need for sustained and consistent professional training among healthcare professionals concerning the identification, documentation, and response to disclosures, suspicions, and allegations of DFV to better support victim-survivors presenting to the ED and other hospital settings.

Global estimates indicate that 30% of women worldwide have experienced some forms of intimate partner violence in their lifetime, with the majority of this abuse (27%) being perpetrated by a partner or ex-partner (World Health Organisation [WHO], 2021). This form of Domestic and Family Violence (DFV) can manifest in various ways, including physical, emotional, psychological, sexual, or financial abuse (Australian Institute of Health and Welfare [AIHW], 2019; WHO, 2021), and it can take place through face-to-face interactions or be facilitated by technology (Dragiewicz et al., 2018). This abuse can cause severe physical health consequences but also leads to long-term psychosocial impacts on victim-survivors who may report significant deterioration in their mental health (Stubbs & Szoeke, 2022; Trevillion et al., 2012).

The health care system is an important point of contact, where victim-survivors can be identified and supported. Research evidence suggests that seeking formal help through avenues like the emergency department (ED) is more common among women subjected to frequent and severe psychological abuse, as well as those who have sustained physical injuries requiring medical care (Fanslow & Robinson, 2010; Meyer, 2010; Tengku Hassan et al., 2015). This initial interaction with health care professionals serves not only to address the current physical or psychosocial issues resulting from DFV, but also as an opportunity in minimizing the risk of future abuse through safety plans and referrals, and counseling (Lewis et al., 2018; Morse et al., 2012).

Despite the clear need for both physical and psychosocial support within the ED victim-survivors express experiencing biased attitudes leading to the provision of suboptimal care from ED clinicians (Hegarty et al., 2020). Women experiencing abuse, and biased attitudes which is reflected in the care provided may subsequently underreporting their abuse (Evans et al., 2016; Fernández-Fontelo et al., 2019), including those presenting to the ED (Dawson et al., 2019). As many women experiencing DFV experience concurrent mental health issues there is a significant vulnerability within victim-survivors.

One way in which poor attitudes and bias may present itself is through the use of language within clinical documentation. Evidence suggests that people’s attitudes can manifest through the language they use to describe phenomena or situations (Lindquist et al., 2015). The language that healthcare providers use in their clinical communication, documentation, and patient notes, construct narratives about patients and their experiences (Healy et al., 2022). A growing body of research involving clinicians has demonstrated that the use of biased or prejudiced language can influence the attitudes and perceptions of individuals reading such documentation. For example, results from a study investigating attitudes associated with drug use terminology showed that medical practitioners report more negative attitudes toward the term “substance abuser” compared to “substance use disorder” (Kelly & Westerhoff, 2010). These findings are consistent with more recent evidence indicating that research participants have significantly more negative automatic attitudes toward the term “substance abuser” compared to “person with a substance use disorder” (Ashford et al., 2019). Reading biased language in medical records, including documentation that introduces doubt, depict the patient negatively, or imply patient responsibility, has been found not only to influence attitudes among health professionals but also to impact the quality-of-care patients receive resulting in decreased help seeking behaviors (Goddu et al., 2019; Keyes et al., 2010).

While a substantial body of literature has examined the association between healthcare professionals’ attitudes and the language used in clinical notes, along with its impact on treatment responses, there remains a gap in understanding how health providers document DFV in the medical records of women presenting to the ED. Given that ED settings are often the initial point of contact for many victim-survivors and considering the potential impact of healthcare professionals’ word choice on staff perceptions, quality of care, and referral pathways for individuals presenting with emergency support needs, it is imperative to examine how ED healthcare providers document DFV. Understanding the ways in which language is used could contribute to developing strategies for the identification of intervention opportunities and the provision of adequate care that aligns with the recovery needs of victim-survivors. In this study, we aim to address this gap by identifying and providing a comprehensive description of the patterns in the language used by ED healthcare providers when documenting instances of DFV in their clinical notes.

## Methods

Qualitative descriptive research is commonly employed to explore phenomena in health and social sciences when additional information is needed for the development or improvement of provision of care or interventions (Kim et al., 2017). This study drew from the existing clinical documentation of a purposeful sample and employed a conventional (inductive) content analysis where the themes were derived from the data (Hsieh & Shannon, 2005) to determine themes and findings in alignment with key elements of qualitative descriptive research (Kim et al., 2017). This approach was utilized to examine the documented language surrounding DFV in the clinical notes of medical charts of women who presented to the ED with mental health concerns where DFV had been identified during their hospital presentation.

### Data Collection

Charts were identified through a search of the Emergency Department Information System. Search parameters included patients that were female patients over the age of 18, who presented to the ED between 1 January 2020 and 1 January 2022 and received referral to the Mental Health Acute Care Team. This timeframe was chosen to facilitate access to physical records as many medical charts are stored off site. A list of over 600 charts were identified meeting these parameters. Considering availability and access issues relating to the physical charts 300 charts were extracted and screened by the research team.

### Data Screening

The research team are drawn from a variety of different epistemological backgrounds including mental health, emergency, criminology, nursing, and social work. This added varying perspectives and understandings to the documentation when viewed through these lenses. The team screened these charts to identify cases where DFV was noted during the ED presentation. Key terms that the team screened for included words such as assault, altercation, punched, incident, etc., this resulted in a final sample of 32 charts included in this study. These encompassed a total of 43 presentations to the ED, as some patients had more than one ED presentation where DFV was identified. All personal identifying information was removed before starting the data entry and analysis phases to preserve patient confidentiality.

### Data Entry

Descriptive qualitative data, specifically handwritten and computerized notes on various hospital forms included in patient charts, were digitized and entered into NVivo 1.7.1 software. The forms included general progress notes, mental health assessment plans, triaging notes, risk screening forms, observation forms etc. Within a standard hospital presentation, it is not uncommon for a substantial amount of clinical documentation to be generated even more so if the patient presents with a high acuity of need. This clinical documentation consisted of many pages (for some patients more than 30 pages) of healthcare related data per patient written by various members of the healthcare team such as nurses, doctors, and psychologists.

### Analysis

The healthcare professionals’ documentation of DFV in clinical notes was coded using an inductive coding approach. A conventional (inductive) content analysis (Hsieh & Shannon, 2005) was used to investigate, without preconceived categories, the language employed by healthcare professionals attending women presenting with DFV at the ED. This approach was selected as it facilitated a qualitative description of the different ways healthcare professionals used language to discuss DFV within their clinical documentation, allowing the emergence of new insights (Kondracki et al., 2002). Two of the authors read the patients’ charts multiple times for data familiarization and coding across the dataset. Throughout, an inductive category application process (Mayring, 2021), data containing references of DFV in the clinical notes were read and coded line-by-line to develop initial codes. These initial codes were reorganized, and a codebook was developed. The authors then analyzed the codes for overlaps in content, context, and use of language through rigorous discussion to arrive at the categories with their definitions and examples from the data. Data extraction and analysis were performed using NVivo 1.7.1 software.

Within the sample, patients had contact with various healthcare staff (e.g., doctors, nurses, social workers, psychologists). As a result, some presentations included multiple documented notes discussing their experience of DFV by different healthcare professionals that could be classified into different themes, resulting in the categories which were not mutually exclusive for each patient or presentation.

### Ethical Considerations

Rigor, trustworthiness, credibility, and researcher reflexivity was considered within the research design and execution. The highly experienced research team was drawn from various specialty backgrounds including emergency nursing, mental health, criminology, and social work. This promoted a robust research design and facilitated expertise in the methodological approaches employed and an understanding of the situatedness and context of the study environment. Patients were not sought for individual consent of their medical charts which are held as the property of the public health system. Due to the research design the contact details of the patients may have been no longer current and furthermore as this study was exploring health presentations relating to intimate partner violence contacting these women outside of a secured place of safety may have presented a risk to themselves. Therefore, to obtain access to these medical charts the research team undertook a rigorous process of ethical review including obtaining ethical approval from the hospital and health service and from the multiple universities involved. As patients were not individually approached for consent the research team was required to apply for Public Health Approval to access each individual chart and each hospital system containing information relevant to the patient’s presentation. This required further approval from each individual system provider. The team then had to provide significant personal data and adhere to strict procedures surrounding the physical movement of this data and ensuring no identifiable data was available outside of the hospital site nor could it be used for secondary purposes. This process of ethical review and approval was conducted over a 12-month timeframe with approval granted by The Prince Charles Hospital Human Research Ethics Committee and Griffith University and The University of the Sunshine Coast (HREC approval number: HREC/2022/TPCH/86965; Public Health Approval: 86965).

## Results

The study included 43 presentations involving 32 biological female patients who visited a regional ED, during which instances of DFV were noted. Transwomen we not specifically excluded but none were identified within the sampling procedures. Women were aged 18 to 56 with a mean age of 32 years. Four categories were identified depicting the manner in which DFV was clinically documented (Table 1). Overall, most of the clinical notes alluding to DFV instances that were coded and analyzed were generated by medical practitioners (e.g., doctors, junior/senior house officers, registrars), followed by nurses, social workers, mental health clinicians (e.g., mental health nurse with advanced practice, psychiatrists, and psychologists), and ambulance officers. The clinical notes referenced a broad spectrum of abusive experiences, encompassing physical violence, emotional and/or verbal abuse, sexual abuse, coercive control, and financial abuse.

**Table 1.** DFV Formulation Categories.

Name	Definition	Example
DFV articulated in direct speech	Formulations based on people’s reports or descriptions of DFV. These can include patient’s reports of absence or presence of DFV, but also other’s reports, such as family or friends	• “she states he [partner] manipulates her into doing what he wants”
		• “[patient] says she has been living in a DV [Domestic Violence] relationship for the last 10 years”
		• “[patient’s father] states that he was sitting outside while [patient] and [partner] were arguing inside, which turned into a physical altercation”
Unambiguous DFV	Violent or abusive incidents are unambiguously formulated by using explicit terminology that clearly describes DFV events and/or dynamics (e.g., abuse, DFV, coercive control, DFV victim, DFV perpetrator)	• “very concerning indicators of D&FV [Domestic and Family Violence] with recent escalation”
		• “hit 5x [five times] by ex-partner”
		• “cycle of DV ongoing issues about the relationship”
Unconfirmed DFV	DFV is carefully articulated, suggesting the potential occurrence of violence incidents, yet certainty remains elusive	• “alleged domestic abuse”
		• “alleged sexual assault last evening”
		• “alleged assault by partner (choke, kick in head)”
Problematic relationship	DFV is formulated in terms of problematic relationship described with a negative connotation but without explicitly identifying DFV dynamics	• “relationship issues”
		• “argument with ex-boyfriend this morning”
		• “identified that there have been ongoing marital issues”

### DFV Articulated in Direct Speech

The most common way in which healthcare professionals described instances of DFV was by articulating it as reported by the patient or by others, such as family members or friends. This type of documentation was observed in 35 out of the 43 ED representations that were reviewed. Overall, the majority of clinical notes including this form of articulation referenced patients’ detailed accounts of their own victimization experiences of physical abuse, verbal and/or emotional abuse, coercive control and financial abuse. For example, one social worker wrote

“She [patient] described DFV [domestic and family violence] tactics including emotional abuse, isolation and gaslighting. Pt [patient] states partner threatens to leave regularly, but feels he stays with her only for financial reasons. She [patient] states he [partner] manipulates her into doing what he wants” (VS11P2).

Further, another social worker noted “Pt [patient] described incidents of strangulation, holding knife to throat, forks or anything he [partner] can get to threaten her” (VS17P1), and a doctor wrote “[patient] reports that he had kicked the car and was spitting at her, she reports that she had called QPS [Queensland Police Service] due to feeling threatened by partner” (VS14P1). Healthcare professionals sometimes quoted patients’ reports of abusive experiences accompanied by the presence of physical violence indicators. For instance, an ambulance officer wrote “Pt [patient] states husband is violent toward her and she states she has bruises on her body from last night’s altercation” (VS22P1), and a social worker noted “Missing teeth and [patient] stated that was from her ex-partner hitting her” (VS25P1).

In addition to direct quotations from patients recounting their victimization experiences, healthcare staff occasionally reported patients’ accounts in a way that could reflect uncertainty about the presence of DFV. For example, a nurse wrote “[Patient] has alluded to the possibility of domestic abuse occurring against her (some gaslighting/emotional manipulation)” (VS32P1). There were instances where healthcare professionals documented patients denying being victims of DFV. For example, one doctor noted “[patient] denies physical harm by partner. States [patient] had harmed self in order to avoid harming partner” (VS19P1). This could reflect patients’ resistance to disclose their victimization experiences to healthcare professions possibly due to fear of not being believed or the potential for further victimization. Lastly, in some cases, documentation of DFV were presented as disclosures by patients’ friends or family members. For instance, an ambulance officer wrote “Friend also concerned pt [patient] had been assaulted by pts [patient’s] ex-partner this evening due to blood on pt [patient’s] clothes” (VS1P1).

### Unambiguous DFV Documentation

In the majority of the reviewed patients’ presentations to the ED (*n* = 32), healthcare professionals used language that described clear instances of abuse. Unambiguous articulations of DFV included references of contexts where DFV was currently occurring or had been recently present. For instance, one nurse wrote “experiencing EDV [emotional domestic violence] from partner” (VS12P4), and another nurse noted “currently living in a DV relationship with husband” (VS11P2). In these examples, the healthcare professionals explicitly identify and acknowledge patients as victim-survivors of DFV. In their notes, some healthcare practitioners clearly articulated experiences of DFV in terms of precursors associated with patients’ presentations to the ED. For example, one doctor wrote “presenting suicidal ideation post an episode of domestic violence” (VS18P1), and a nurse noted “Last night deliberately drove her car off an embankment into a tree with the intent to end her life due to many psycho-social events including living in a DV relationship” (VS11P2).

Similarly, several clinical notes included references to the patient as a victim-survivor by either using terms such as “victim” or explicitly identifying the patient’s partner as violent or abusive. For example, one nurse wrote “victim of DV socially isolated” (VS12P1), and a doctor noted “[patient] lives with violent partner” (VS6P2). While healthcare providers did not always use the terms “DFV” or “DV” in their clinical notes, some employed unambiguous depictions of abuse while reporting patients’ presentations. For example, one psychiatrist recorded “she took an overdose while intoxicated in the context of being harassed by her ex-partner” (VS10P1). Some clinical notes also included specific descriptions of physical violence, exemplified by a note from an ambulance officer stating, “[patient] assaulted by partner kicking her in the head & trying to strangle her” (VS6P2).

In addition to current DFV incidents, a history of abuse was cited in approximately a third (*n* = 14) of the examined ED presentations, with nurses being the healthcare professionals who most referenced past instances of DFV. For example, one nurse wrote, “DV incident in August, perpetrator of DV was ex-partner” (VS2P1), and another one, “At risk of victimization due to history of DV” (VS25P1). The latter example demonstrates the utility of healthcare professionals referring to patients’ history of DFV in assessing potential risks and creating opportunities for prevention.

### DFV Articulated as Unconfirmed Events

In nearly half of the ED presentations (*n* = 17), healthcare providers documented DFV incidents as unconfirmed events. This form of DFV documentation was most frequently employed by doctors. Caution was exercised when discussing assertions regarding patients’ victimization, suggesting that accusations against a partner or ex-partner were raised, but these accounts had not been substantiated or might be under investigation. Examples include “Alleged assault by ex-partner” (VS17P1) and “pt [patient] presenting to ED following alleged assault by partner” (VS6P2).

Similar documentation of DFV was also documented when reporting detailed accounts of clinical indicators, such as physical effects of abuse, or heightened arousal resulting from the assault. For example, a nurse wrote, “alleged assault tonight. 13 weeks gestation, kick to stomach. Pain to occipital and side of neck with abrasions to L [left] forearm, lower back, and throat and jaw” (VS8P1). Another nurse noted, “Pt [patient] startling easily when touched, concerned person who allegedly assaulted her was present in room” (VS1P1). The use of legal language by healthcare professionals in documenting DFV incidents suggests an acknowledgment that the patient might have been a victim of DFV. However, as the events remain unconfirmed, precaution might be taken to mitigate potential legal repercussions. Some clinical notes indicated a degree of suspicion or doubt about the occurrence of DFV, as evidenced by the description of the abusive event being preceded by a question mark or terms such as “possible.” Examples include “? post assault by partner” (VS21P1) and “for ? DV related issues’’ (VS5P1).

### DFV as Part of Problematic Relationships

About half of the examined ED presentations (*n* = 22) featured clinical notes in which DFV instances were characterized within the context of relationship “problems,” “issues” or “arguments,” often framed as mutual or reciprocal behavioral dynamics between the patient and their partner or ex-partner. Overall, although all healthcare professionals used this type of documentation, doctors described DFV in terms of relationship problems more often. One ambulance officer noted, “patient having relationships problems” (VS11P2), and a psychiatrist wrote, “[patient] has presented with deterioration of mental state and increasing suicidality, in the context of ongoing psychosocial stressors including relationship issues with partner/father of children” (VS12P2). This language, unlike terminology describing abuse, might convey a tone of trivialization or suggest an underestimation of patients’ experiences of victimization. Similarly, a doctor noted, “Patient has been fighting with ex-husband” (VS10P1), and a nurse wrote “argument with partner and self-harm” (VS14P1). By using terms such as “fighting” or “arguments”, healthcare providers create an impression of DFV being bi-directional. This hinders efforts to identify the primary victim-survivor and aggressor in the relationship, which can lead to an underestimation of the risks that these situations pose to the patient. This, in turn, could affect the quality and adequacy of care that patients receive.

Some relational dynamics that were captured in healthcare workers’ clinical notes were not described as abusive or violent but, instead, categorized as “unhealthy,” “turbulent,” or “toxic.” For example, a social worker noted, “marriage is toxic” (VS16P1) and a mental health clinician wrote, “Relays unhealthy relationship where [partner] argumentative and yelling at her” (VS3P1). Although these descriptions of the relationship dynamics between patients and their partners carry a negative connotation, the use of such language minimizes the severity of patients’ experiences of victimization and related risk of harm.

## Discussion

This article sought to understand how healthcare professionals document DFV in the medical records of women presenting to the ED. The results of this study suggest that, while the majority of DFV references found in the medical charts directly quoted instances of abuse or were documented unambiguously by healthcare professionals, a significant number of clinical notes indicated a degree of caution or lack of acknowledgment of DFV dynamics when describing such events.

The most common method employed by healthcare professionals in reporting DFV in clinical notes was through direct quotations from patients, family members, and friends. Several reasons may underlie the use of direct quotations. For instance, healthcare providers may use patients’ exact wording to accurately document events in a neutral fashion, aiming to preserve objectivity and prevent misinterpretation. Documenting patients’ self-reports concerning their ED presentation can assist in amplifying the patients’ own voice. Research suggests that healthcare services, including EDs, are often the initial point of contact for victim-survivors (Koistinen & Holma, 2015), and women experiencing DFV are more likely to use ED services at a higher rate than other women (Olive, 2007). Thus, accurately documenting women’s presentations and disclosures of DFV in their own words is important to formally record their victimization experiences. However, it is well-established in the literature that women tend to underreport their experiences of DFV (Evans et al., 2016; Fernández-Fontelo et al., 2019; Gracia, 2004), and research indicates that patients are reluctant to disclose these experiences to professionals within the ED setting (Dawson et al., 2019; Yam, 2000). For this reason, depending solely on patients’ self-reports during their ED evaluations may result in an underestimation of the significance of DFV in the context of their presentations. Therefore, when healthcare professionals suspect a patient might be a victim-survivor of DFV, even without explicit disclosure, it is crucial that they document their observations and concerns regarding potential abuse.

Indications of suspicion regarding DFV were frequently observed in the reviewed medical charts. However, this documentation was not consistently accompanied by a rationale supporting the concern for possible abuse. Further, results showed that in nearly half of the reviewed cases, healthcare providers used words or punctuation marks such as “allegedly,” “possible,” or question marks. While the presence of this language pattern does not unequivocally deny patients’ victimization experiences, it might suggest doubt or uncertainty. This type of documentation of DFV raises concerns, particularly in cases where healthcare providers do not seek additional information. Healthcare professionals’ inaction often result from lack of guidelines or knowledge regarding DFV (WHO, 2013). For example, results from a study by Sargeant et al. (2023) indicate a lack of confidence regarding DFV screening and tensions among front-line clinicians initiating conversations about DFV while managing their own stress. Additional barriers to DFV identification have been recognized in the literature, including lack of time, training, privacy, guidelines, policies, support from the employer and ED professionals’ inappropriate beliefs (Fisher et al., 2020; Hinsliff-Smith & McGarry, 2017; Kirk & Bezzant, 2020; Yonaka et al., 2007). In addition, assumptions regarding role responsibilities can further hinder the assessment and subsequent documentation of abuse. For instance, results from a qualitative study by Dawson et al. (2019) showed that clinical staff in the ED acknowledged the role of nurses in actively pursuing and verifying their suspicions of DFV. However, according to the participating nurses, the responsibility for investigating potential and confirmed cases of abuse lies with social workers. This belief might explain why doctors, as opposed to social workers, most frequently made DFV documentation categorized under “unconfirmed DFV” in our study. However, nurses and social workers expressed doubt in their DFV documentation at a similar rate. Considering the crucial role of clinical notes in healthcare communication, results from this study emphasize the need for increased efforts, particularly among doctors, in documenting and further investigating suspicions of abuse. These enhancements could subsequently contribute to improvements in healthcare responses.

Some examples reported in this article illustrate cases in which patients’ presentations to the ED were framed within the context of abuse by a partner or ex-partner, providing greater insight into the patients’ situation. Unambiguous documentation of DFV validate and legitimize patients’ experiences as victim-survivors, potentially reducing existent distrust toward healthcare professionals (Wallin Lundell et al., 2018). Further, by incorporating victimization experiences as a key element when describing women’s ED presentations, healthcare professionals can enhance their responses to patients’ care needs. This involves addressing not only physiological or psychological health needs but also psychosocial ones. Explicit mentions to patients’ history of DFV victimization can aid in assessing and identifying current and future potential risks, as well as in creating opportunities for addressing psychosocial impacts and preventing further victimization.

While most of the examined clinical charts provided explicit descriptions of abuse within the context of DFV, approximately half of them depicted DFV characterized as “relationships problems” or issues without evidence of further exploration of the relationship dynamics and related risk. Describing instances of abuse as “conflicts” between the patient and their partner may minimize the perceived severity of the abusive experience and underestimate the risk that the situation poses to the patient. Research suggests that individuals’ attitudes and perceptions can manifest through the way they use language (Lindquist et al., 2015). Accordingly, the terminology describing women’s experiences of DFV as constituting “problematic relationships” may suggest that healthcare professionals fail to recognize and document the severity of the abuse. Moreover, such depictions of violence may be misconstrued as bi-directional or situational couple violence (Johnson, 2006), emerging within the context of specific conflicts that can escalate into violence. This use of language implies that both parties engage simultaneously in abusive behavior toward each other. Documentation that describes the patient as a responsible agent of violence within the relationship might lead to an oversight in recognizing key elements within DFV dynamics, such as the existing imbalance of power and a motivation for control (Stark, 2009). Further, it can result in the misidentification of the patient as the perpetrator, overlooking the patient’s care and support needs.

Receiving timely and specialized care has been identified as a factor contributing to positive patient experiences in the ED (Duchesne et al., 2023). Therefore, it is crucial that professionals working in the ED adopt a trauma-informed approach, enabling a comprehensive and holistic response to the needs of victim-survivors’ (Huo et al., 2023). Trauma-informed approaches should include not only what and how a healthcare professional verbally discusses DFV with the victim-survivor but also how they document care and holistically consider the needs of their patient (Huo et al., 2023). Lastly, it is crucial to provide adequate training and establish clear guidelines for emergency health care workers on how to report DFV-related findings. This guideline in alignment with trauma-informed care approaches should consider not only physical care but also attend to other needs such as social support, safety and security, autonomy, emotional well-being or financial stability (Huo et al., 2023). Such measures would ensure consistency in the approach to DFV across professionals and the accurate documentation of the nature and severity of the violence experienced by patients.

### Limitations

There are several limitations to the present study. First, the data were collected from clinical notes relating to presentations to the ED, which may limit the generalizability of these findings to other hospital settings. Furthermore, the data was collected from a single regional hospital within Australia and therefore one should consider the transferability of these results and recommendations to other geographical areas. Lastly, the analysis did not consider patients’ individual characteristics, such as ethnicity or sexual orientation or gender identity. These demographic characteristics may be important factors associated with how healthcare professionals describe and perceived patients’ experiences of DFV. Thus, further research is warranted to investigate whether DFV documentation vary across different patient populations.

## Conclusions

The main goal of medical records is to document clinical information with the purpose of patient management and effective communication with other healthcare professionals. These clinical charts should contain sufficient and accurate information to enable other professionals to take over the record and ensure that patients’ care and support needs are successfully met. Additionally, documentation of DFV within clinical records can be later used as evidence in legal proceedings. Therefore, accurate and comprehensive documentation of DFV is paramount. The findings of this qualitative study indicated significant variability and a lack of trauma-informed language used by healthcare providers in their descriptions of DFV. While most of the reviewed clinical charts included notes where DFV was unambiguously documented, a considerable number of articulations of DFV expressed a degree of doubt or were described as “relationship problems.” Language plays an important role in shaping subsequent healthcare professionals’ attitudes and behavior (Park et al., 2021). The absence of certainty or language that denotes severity in healthcare providers’ descriptions of DFV might have the potential to negatively impact the quality of care provided to women experiencing DFV. This concern becomes particularly relevant when exclusive attention is given to physical indicators in cases involving abuse. Findings therefore support the need for consistent and ongoing professional development in the provision of trauma-informed care specifically around recognizing, documenting, and responding to disclosures, suspicions, and allegations of DFV for healthcare practitioners to improve outcomes for victim-survivors accessing ED and other hospital settings.
